# Quinovic Acid Enhances the Cytotoxicity of KHYG‐1 Cells by Modulating the Ras/MAPK Signalling Pathway and Interferon‐Gamma Expression

**DOI:** 10.1111/jcmm.70957

**Published:** 2025-11-26

**Authors:** Ming‐Ju Hsieh, Jen‐Tsun Lin, Yi‐Ching Chuang, Hsin‐Yu Ho, Yu‐Sheng Lo, Chia‐Chieh Lin, Mu‐Kuan Chen

**Affiliations:** ^1^ Oral Cancer Research Center Changhua Christian Hospital Changhua Taiwan; ^2^ Graduate Institute of Clinical Medicine, College of Medicine National Chung Hsing University Taichung Taiwan; ^3^ Doctoral Program in Tissue Engineering and Regenerative Medicine, College of Medicine National Chung Hsing University Taichung Taiwan; ^4^ Graduate Institute of Biomedical Sciences China Medical University Taichung Taiwan; ^5^ Division of Hematology and Oncology, Department of Medicine Changhua Christian Hospital Changhua Taiwan; ^6^ Department of Otorhinolaryngology, Head and Neck Surgery Changhua Christian Hospital Changhua Taiwan; ^7^ Department of Post‐Baccalaureate Medicine, College of Medicine National Chung Hsing University Taichung Taiwan

**Keywords:** granzyme B, MAPK signalling, natural killer cells, quinovic acid

## Abstract

Quinovic acid is a key constituent of cat's claw (*Uncaria tomentosa*) extract and exhibits antioxidant and anti‐inflammatory activities. In this study, we investigated the potential of quinovic acid to enhance natural killer (NK) cell activity by using the KHYG‐1 cell line. Our data indicated that quinovic acid increased the expression levels of cytolytic molecules, including perforin, granzymes A and B, Fas ligand, and granulysin, and induced the phosphorylation of the transcription factors CREB and STAT4, thereby enhancing cytotoxic activity against K562 cells. Furthermore, when KHYG‐1 cells were cocultured with K562 cells in the presence of quinovic acid, we observed an increase in the expression of t‐Bid, cleaved caspases 3, 8, and 9, and PARP, promoting apoptosis in K562 cells. Quinovic acid also reduced the expression of SET, Ape1, and HMGB2, effectively inhibiting the DNA repair mechanism in target cells. Similar results were observed in other cancer cell lines. In addition, quinovic acid induced interferon‐gamma secretion by upregulating the Ras/MAPK and PI3K/AKT/mTOR signalling pathways through the activation of NKG2D and other NK receptors. These effects were observed not only in KHYG‐1 cells but also in NK cells derived from adult patients with head and neck squamous cell carcinoma. Our findings suggest that quinovic acid enhances NK cell cytotoxicity, showing promise as a potential therapeutic against various cancer cell types.

**Abbreviations**
AKT: protein kinase B
CREB: cAMP response element binding protein
MAPK: mitogen‐activated protein kinases
mTOR: mammalian target of rapamycin
NKRs: natural killer receptors
PARP: poly (ADP‐ribose) polymerase
PI3K: phosphatidylinositol 3‐kinase
STAT: signal transducer and activator of transcription

## 1 Introduction

Natural plant‐derived compounds, such as diterpenes, diterpene quinones, lactone sesquiterpenes, and alkaloids, are a valuable source of anticancer drugs [1]. These compounds enhance the antitumor response of immune cells. For example, curcumin increased the cytotoxic activity of NK92 cells against K562 cells [2]. Resveratrol stimulates interferon‐gamma (IFN‐g) secretion in human natural killer (NK) cells [3, 4]. Our previous studies have demonstrated that shuterin and raddeanin A enhanced cytotoxicity in KHYG‐1 cells by modulating the MAPK and Ras/Raf signalling pathways [5, 6]. Quinovic acid, a naturally occurring triterpene isolated from various plants [7, 8], is a key constituent of cat's claw (*Uncaria tomentosa*) extract. Quinovic acid has been widely used to treat inflammatory disorders because of its anti‐inflammatory and antioxidant properties [9]. A recent study reported that quinovic acid strongly suppressed human breast and lung cancer cell growth and viability, indicating its potent anticancer activity [10]. However, the potential immune‐boosting effect of quinovic acid remains unclear.

NK cells are an essential component of the immune network because of their innate ability to eliminate tumour cells [11, 12]. Upon activation, NK cells release cytotoxic granules, such as perforin and granzymes, that directly lyse tumour cells. In addition, NK cells can eliminate target cells by activating death ligands, such as the Fas ligand (FasL), and by producing chemokines and cytokines, including IFN‐γ and tumour necrosis factor‐alpha (TNF‐α) [13, 14].

KHYG‐1 is a human, permanent NK cell line characterised by its stable expansion and maintenance in vitro. This cell line exhibits stronger cytotoxicity against K562 cells than NK‐92, YT, and SNT‐8 cells, expresses NK receptors, and closely resembles human NK cells. These features make the KHYG‐1 cell line suitable as a model for studying NK cell activity [15, 16, 17, 18]. Our previous study demonstrated that raddeanin A enhances the cytotoxicity of KHYG‐1 cells and expression of NK receptors [5]. In the present study, we elucidated mechanisms through which quinovic acid potentiates NK activity in KHYG‐1 cells.

## 2 Materials and Methods

### 2.1 Chemicals and Reagents

**FIGURE 1:**
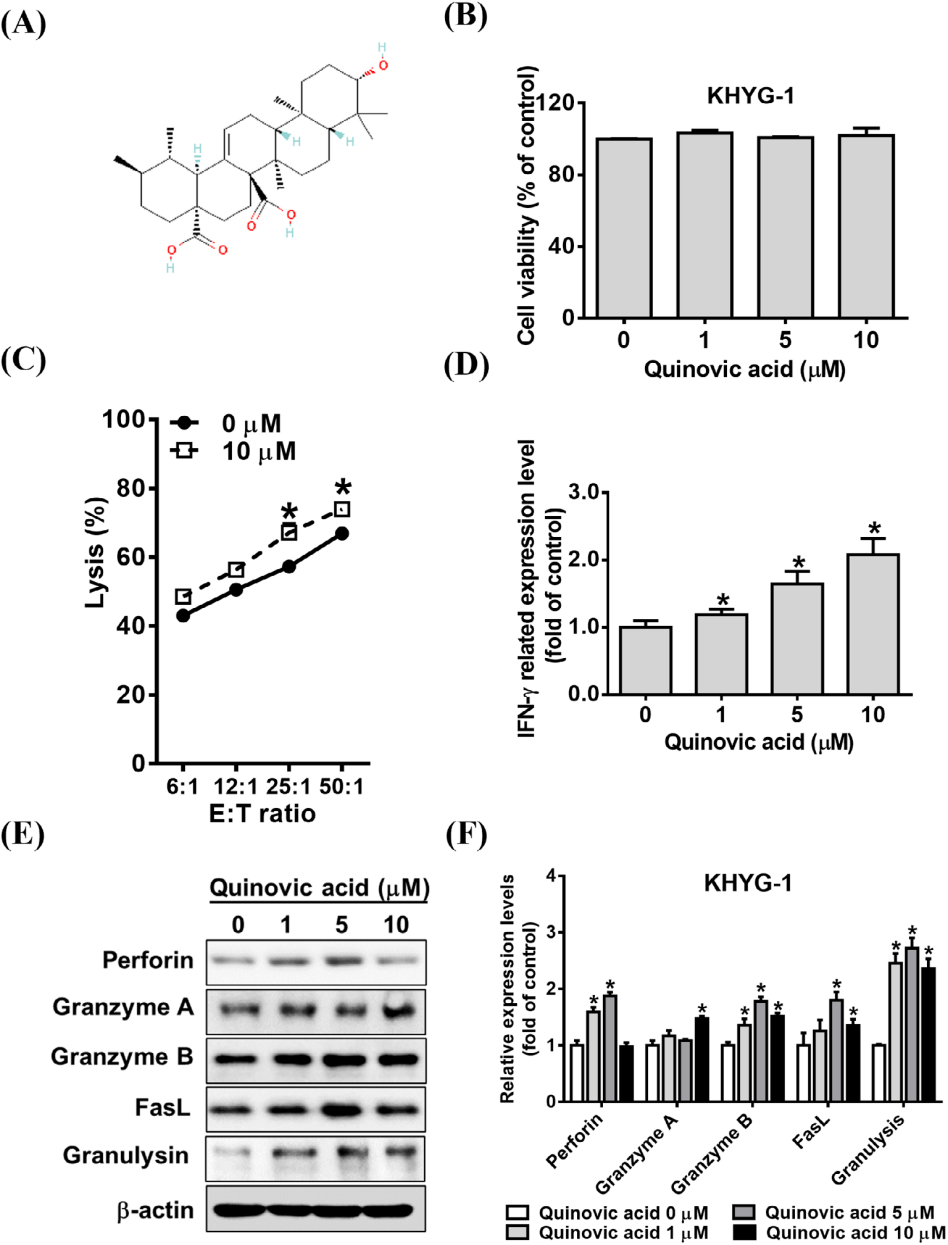
Quinovic acid enhanced the cytolytic effect of KHYG‐1 cells. (A) Chemical structure of quinovic acid. (B) WST‐8 assay results showing the viability of KHYG‐1 cells treated with quinovic acid for 72 h. (C) Calcein AM release assay results demonstrating the cytotoxicity of KHYG‐1 cells treated with quinovic acid against K562 cells for 72 h. (D) Enzyme‐linked immunosorbent assay results showing the IFN‐γ level in the culture supernatants of KHYG‐1 cells treated with varying concentrations of quinovic acid (1, 5, and 10 μM) for 24 h. (E) Western blot analysis of cytotoxicity‐related proteins in KHYG‐1 cells treated with quinovic acid (0–10 μM) for 24 h. (F) Protein quantification was performed using ImageJ; all protein levels were normalised to that of β‐actin. Data are presented as mean ± standard deviation from three independent experiments. *p < 0.05 compared with control.

Quinovic acid (purity ≥ 98%; Figure 1A) was purchased from ChemFaces (Wuhan, China). Stock solutions were prepared in dimethyl sulfoxide (DMSO) and stored at −20°C. All drug treatments were conducted using a DMSO concentration of < 0.1%. Calcein AM was obtained from AAT Bioquest. The histone deacetylase inhibitor trichostatin A (TSA) and the granzyme B inhibitor Z‐AAD‐CMK were purchased from Sigma‐Aldrich (St Louis, MO, USA). Specific inhibitors of U0126 and SB203580 were obtained from Santa Cruz Biotechnology (Santa Cruz, CA, USA). Antibodies were purchased from Cell Signalling Technology (Danvers, MA, USA) and stored at −20°C. β‐actin was bought from Novus Biologicals (Centennial, CO, USA).

### 2.2 Cell Culture

The human NK leukaemia cell line KHYG‐1 (accession no. JCRB0156) and the human chronic myelogenous leukaemia cell line K562 (accession no. JCRB0019) were obtained from the Japanese Collection of Research Bioresources (Japan). KHYG‐1 cells were cultured in Roswell Park Memorial Institute (RPMI‐1640) medium (Life Technologies, Grand Island, NY, USA) supplemented with 10% fetal bovine serum (Merck Millipore, Burlington, MA, USA), 100 U/mL recombinant human interleukin (IL)‐2 (Cat. #200‐02; PeproTech), 100 U/mL penicillin G, and 100 μg/mL streptomycin sulfate. K562 cells were cultured in the same medium without IL‐2 supplementation. All cultured cells were incubated at 37°C under 5% CO_2_. NK cells were isolated from the peripheral blood of adult donors with head and neck squamous cell carcinoma. Peripheral blood mononuclear cells were cultured using BINKIT devices (Biotherapy Institute of Japan, Tsukuba, Japan), in accordance with the manufacturer's instructions. The subculture medium contained 10% heat‐inactivated autologous plasma and 400 U/mL recombinant human IL‐2. All experiments were conducted within 21 days of culture.

### 2.3 Cell Proliferation Assay

Cell proliferation was assessed using the WST‐8 assay (Beyotime Technology, Shanghai, China). KHYG‐1, K562, NPC‐039, and FaDu cells were seeded in 96‐well plates at a density of 5 × 10^4^ cells per well, treated with various concentrations of quinovic acid (1, 5, and 10 μM), and cultured with IL‐2 for 24 or 72 h. The absorbance of the formazan product was measured at 450 nm by using an enzyme‐linked immunosorbent assay (ELISA) plate reader to determine cell proliferation.

### 2.4 Cell Cytotoxicity Assay

The cytotoxic effect of quinovic acid was evaluated using a calcein AM release assay as described elsewhere [19]. KHYG‐1 cells (effector cells) were treated with the indicated doses of quinovic acid for 72 h. K562 cells (target cells) were stained with 10 μM calcein AM for 30 min at 37°C under 5% CO_2_ in complete RPMI 1640 medium. After staining, KHYG‐1 and K562 cells were cocultured in 96‐well plates for 2 h (without additional quinovic acid). Fluorescence intensity was measured at an excitation/emission wavelength of 485/530 nm. Percent lysis was calculated using the following formula: [(experimental release − spontaneous release)/(maximum release − spontaneous release)] × 100%.

### 2.5 Cytokine Secretion

KHYG‐1 cells were treated with the indicated dose of quinovic acid for 24 h and then centrifuged at 190× *g* for 5 min at 4°C. The supernatant was collected to measure the IFN‐γ level by using a LEGEND MAX Human IFN‐γ ELISA kit (BioLegend, San Diego, CA, USA), following the manufacturer's instructions.

### 2.6 Western Blot Analysis

KHYG‐1 cells were treated with the indicated doses of quinovic acid for 24 h, and Western blot analysis was performed as described elsewhere [20]. K562, NPC‐039, and FaDu cells were cocultured with KHYG‐1 cells for 24 h before collection. Cells were lysed using RIPA buffer, and equal amounts of protein were separated through sodium dodecyl sulfate‐polyacrylamide gel electrophoresis (SDS‐PAGE) and transferred onto a polyvinylidene fluoride membrane (Merck Millipore, Burlington, MA, USA). Membranes were blocked for 1 h with 5% nonfat milk in TBST and then incubated overnight at 4°C with the indicated primary antibodies. Subsequently, membranes were incubated at room temperature with the appropriate peroxidase‐conjugated secondary antibodies, and bands were visualised using an electro‐chemiluminescent detection system. Protein quantification was performed using ImageJ.

### 2.7 Mitochondrial Membrane Potential Measurement

KHYG‐1 cells were treated with the indicated doses of quinovic acid and added to the upper wells of a Transwell insert (Greiner Bio‐One, Monroe, NC, USA). K562, FaDu, or NPC‐039 cells in RPMI‐1640 medium were placed in the lower wells of the same insert and incubated following previously described coculture methods [5]. The effector (KHYG‐1) and target cells were cocultured at an effector‐to‐target ratio of 6:1 for 24 h at 37°C under 5% CO_2_. After incubation, the target cells were collected, stained with Muse MitoPotential dye for 20 min at 37°C, and incubated for 5 min with 7‐AAD. Experimental signals were measured using a Muse Cell Analyser (Merck Millipore, Burlington, MA, USA), and data were analysed using Muse Cell Soft V1.4.0.0.

### 2.8 Annexin V and Propidium Iodide Double Staining

Apoptosis detection using annexin V and propidium iodide (PI) double staining was performed as described previously [21]. KHYG‐1 cells were treated with the indicated doses of quinovic acid and placed in the upper wells of a Transwell insert (Greiner Bio‐One, Monroe, NC, USA). Target cells (K562, FaDu, and NPC‐039) were added to the lower wells of the same insert and incubated following previously described coculture methods [6]. The effector (KHYG‐1) and target cells were cocultured at an effector‐to‐target ratio of 6:1 for 24 h at 37°C under 5% CO_2_. After incubation, the target cells were collected, resuspended in annexin V/PI double staining buffer (Millipore), and incubated for 20 min at room temperature in the dark. The stages of apoptosis were measured using a Muse Cell Analyser (Merck Millipore, Burlington, MA, USA), and data were analysed using Muse Cell Soft V1.4.0.0 Analyser.

### 2.9 S tatistical Analysis

Statistical analyses were performed using GraphPad Prism Software Version 5.0 (GraphPad Software, La Jolla, CA, USA). Each experiment was conducted at least three times, and data are presented as the mean ± standard deviation (SD). One‐way analysis of variance followed by Tukey's multiple comparisons test was used to assess differences between groups (control and various drug doses). A *p* value of < 0.05 was considered statistically significant.

## 3 Results

### 3.1 Effect of Quinovic Acid on the Cytotoxicity of KHYG‐1 Cells Against K562 Cells

Figure 1A presents the chemical structure of quinovic acid. Initially, KHYG‐1 cells were treated with different concentrations of quinovic acid (1, 5, and 10 μM) for 72 h, and cell proliferation was analysed using the WST‐8 assay. We observed no regulatory effect of quinovic acid on cell viability and proliferation in the control and treatment groups (Figure 1B). Next, we examined the effect of quinovic acid treatment for 72 h on the cytotoxicity of KHYG‐1 cells against K562 cells by using the calcein AM release assay. The results revealed that treatment with quinovic acid significantly increased the cytotoxicity of KHYG‐1 cells (Figure 1C).

A study indicated that IFN‐γ enhances the antitumor and antiviral effects of NK cells [22]. Thus, we examined the effect of quinovic acid on IFN‐γ secretion in KHYG‐1 cells. We observed that IFN‐γ secretion by KHYG‐1 cells significantly increased with the concentration of quinovic acid (Figure 1D). In addition, we performed Western blot analysis to examine the expression of cytotoxicity‐related proteins. The results indicated that quinovic acid treatment increased the expression of perforin, granzyme A, granzyme B, FasL, and granulysin (Figure 1E,F).

### 3.2 Effects of Quinovic Acid on Transcription Factors and Acetyl Histone Expression

**FIGURE 2:**
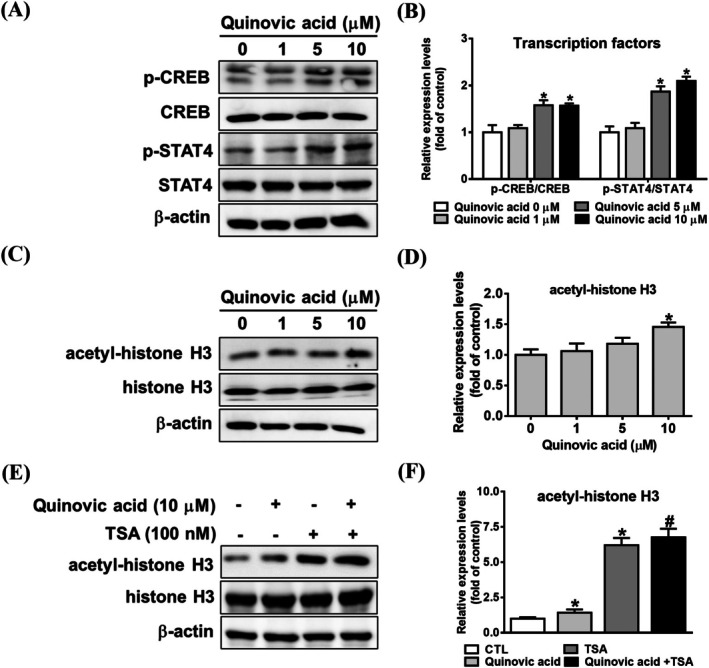
Quinovic acid increased transcription factor activity and affected histone acetylation. KHYG‐1 cells were treated with quinovic acid (0–10 μM) for 24 h. Western blot analysis was performed to determine protein levels of phosphorylated CREB/total CREB, phosphorylated STAT4/total STAT4 (A, B), and acetyl‐histone H3/total histone H3 (C, D). In a separate experiment, cells were pretreated with TSA for 1 h, followed by cotreatment with quinovic acid (10 μM). Proteins were collected after 24 h to analyse acetyl‐histone H3/total histone H3 levels (E, F). Protein quantification was performed using ImageJ, with all protein levels normalised to that of β‐actin. Data are presented as mean ± standard deviation from three independent experiments. *p < 0.05 compared with control; ^#^ *p* < 0.05 compared with TSA alone.

Our results indicated that quinovic acid significantly increased granzyme B and INF‐γ expression in KHYG‐1 cells. Phosphorylated CREB regulates granzyme B protein expression [23]. The STAT4 signalling pathway is crucial for IFN‐γ transcription in NK cells [24, 25]. Thus, we investigated the effect of quinovic acid on the phosphorylation of CREB and STAT4 transcription factors. Quinovic acid treatment increased the phosphorylation levels of CREB and STAT4 in KHYG‐1 cells (Figure 2A,B). In addition, we examined the expression of acetylated histone H3 (Lys9/Lys14) and determined whether the effect of quinovic acid on this expression was enhanced when combined with TSA treatment (Figure 2C–F). Our results revealed that quinovic acid increased the expression of acetylated histone H3 (Lys9/Lys14), and this effect was more significant when combined with TSA.

### 3.3 Effect of Quinovic Acid on Apoptosis Signalling Pathways

**FIGURE 3:**
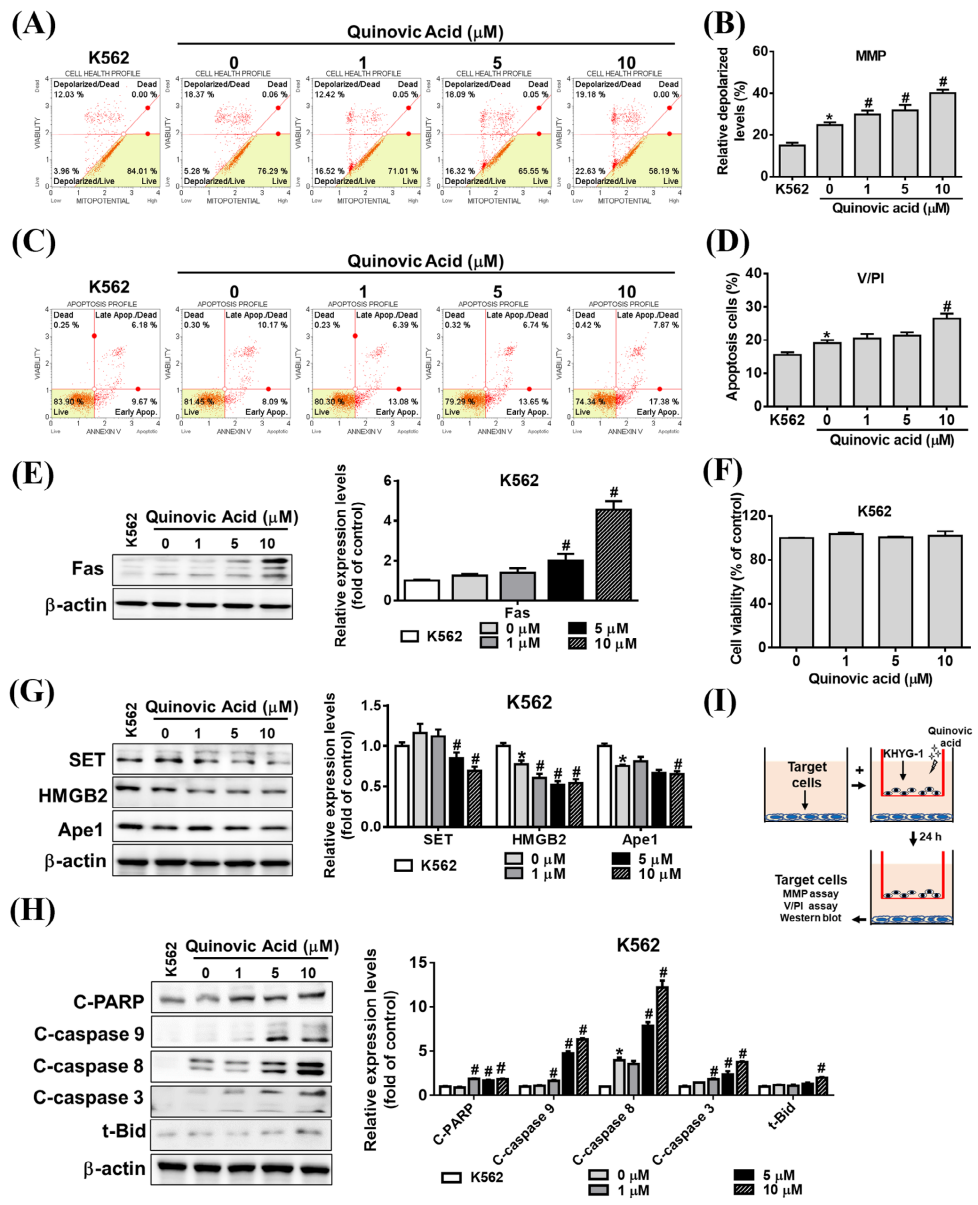
Analysis of quinovic acid‐induced apoptosis in KHYG‐1 cells cocultured with K562 cells. KHYG‐1 cells were cocultured with K562 cells at an effector‐to‐target ratio of 6:1 and treated with quinovic acid (0–10 μM) for 24 h. (A, B) Mitochondrial membrane potential assay. (C, D) Annexin V and PI staining of K562 cells, measured using a Muse Cell Analyser. Quantitative data were analysed using Muse Cell Software V1.4.0.0. Western blot analysis was performed to assess protein levels associated with quinovic acid‐induced apoptosis. (E) Fas protein expression. (F) WST‐8 assay results assessing K562 cell viability following treatment with quinovic acid (0–10 μM) for 24 h. (G) Proteins related to the SET complex: SET, HMGB2, and Ape1. (H) Caspase pathway–related proteins and t‐Bid. Protein quantification was performed using ImageJ, with all protein levels normalised to that of β‐actin. (I) Schematic diagram of the coculture model. Data are presented as the mean ± standard deviation from three independent experiments. *p < 0.05 compared with control; ^#^ *p* < 0.05 compared with cotreatment control.

To elucidate the mechanism through which quinovic acid increases the cytotoxicity of KHYG‐1 cells against K562 cells, we first confirmed that quinovic acid alone does not have a direct cytotoxic effect on K652 cells (Figure 3F). KHYG‐1 cells were then cocultured with K562 cells (Figure 3I) at an effector‐to‐target cell ratio of 6:1 for 24 h. Quinovic acid treatment increased the proportion of depolarised cells in a dose‐dependent manner (Figure 3A,B). Significant differences were noted in FITC annexin V and PI double‐staining results between the treatment and control groups (Figure 3C,D). Next, we examined the effect of cytotoxicity‐related proteins secreted by KHYG‐1 cells on both caspase‐dependent and caspase‐independent apoptotic pathways in cocultured K562 cells. Quinovic acid significantly increased the expression of Fas, the cleaved forms of t‐Bid, caspases 3, 8, and 9 and poly (ADP‐ribose) polymerase (PARP; Figure 3E,H). In addition, quinovic acid significantly reduced the expression of proteins targeted by granzyme A, such as SET, Ape1, and HMGB2 (Figure 3G). Taken together, these findings indicate that quinovic acid enhances KHYG‐1–mediated cytotoxicity, leading to caspase‐dependent and ‐independent apoptotic signalling in cocultured K562 cells.

**FIGURE 4:**
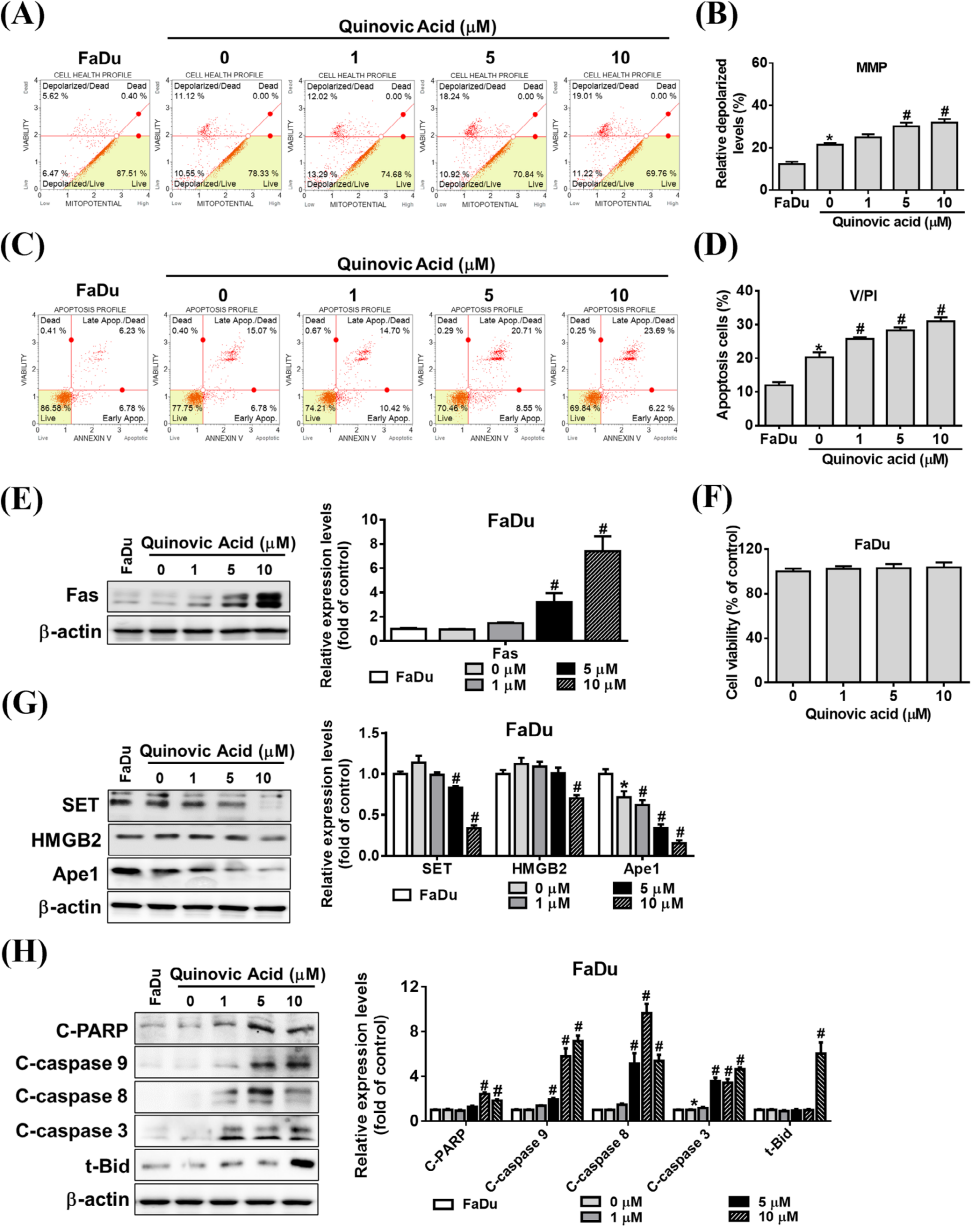
Analysis of quinovic acid‐induced apoptosis in KHYG‐1 cells cocultured with FaDu cells. KHYG‐1 cells were cocultured with FaDu cells at an effector‐to‐target ratio of 6:1 and treated with quinovic acid (0–10 μM) for 24 h. (A, B) Mitochondrial membrane potential assay. (C, D) Annexin V and PI staining of FaDu cells measured using a Muse Cell Analyser. Quantitative data were analysed using Muse Cell Software V1.4.0.0. Western blot analysis was performed to assess protein levels associated with quinovic acid–induced apoptosis. (E) Fas protein expression. (F) Results of the WST‐8 assay assessing FaDu cell viability following treatment with quinovic acid (0–10 μM) for 24 h. (G) Proteins related to the SET complex: SET, HMGB2, and Ape1. (H) Caspase pathway–related proteins and t‐Bid. Protein quantification was performed using ImageJ, with all protein levels normalised to that of β‐actin. Data are presented as the mean ± standard deviation from three independent experiments. *p < 0.05 compared with control; ^#^ *p* < 0.05 compared with cotreatment control.

**FIGURE 5:**
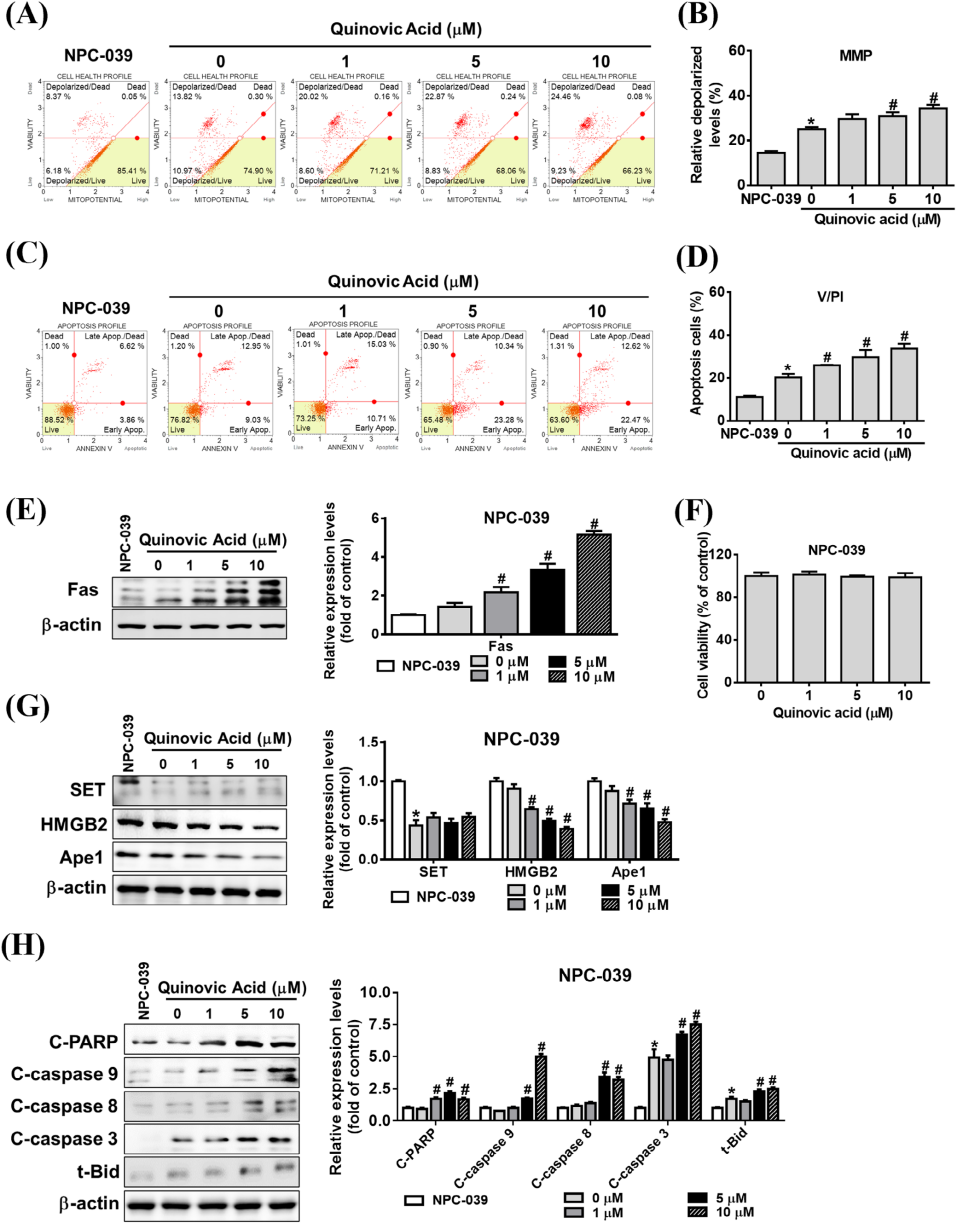
Analysis of quinovic acid–induced apoptosis in KHYG‐1 cells cocultured with NPC‐039 cells. KHYG‐1 cells were cocultured with NPC‐039 cells at an effector‐to‐target ratio of 6:1 and treated with quinovic acid (0–10 μM) for 24 h. (A, B) Mitochondrial membrane potential assay. (C, D) Annexin V and PI staining of NPC‐039 cells measured using a Muse Cell Analyser. Quantitative data were analysed using Muse Cell Software V1.4.0.0. Western blot analysis was performed to assess protein levels associated with quinovic acid–induced apoptosis. (E) Fas protein expression. (F) Results of the WST‐8 assay performed to evaluate NPC‐039 cell viability following treatment with quinovic acid (0–10 μM) for 24 h. (G) Proteins related to the SET complex: SET, HMGB2, and Ape1. (H) Caspase pathway–related proteins and t‐Bid. Protein quantification was performed using ImageJ, with all protein levels normalised to that of β‐actin. Data are presented as the mean ± standard deviation from three independent experiments. *p < 0.05 compared with control; ^#^ *p* < 0.05 compared with cotreatment control.

Under the coculture conditions of KHYG‐1 and K562 cells, quinovic acid promoted the apoptosis of K562 cells. To further evaluate the effects of quinovic acid, we conducted comparative experiments by using two other cancer cell lines, namely the squamous cell carcinoma cell line FaDu and the nasopharyngeal carcinoma cell line NPC‐039. We observed that quinovic acid at the highest tested dose did not exert a cytotoxic effect on FaDu and NPC‐039 cells over a 24‐h period; we used the same dose as that in K562 cells (Figures 4F and 5F). Quinovic acid significantly and dose‐dependently enhanced KHYG‐1 cell‐mediated mitochondrial membrane depolarisation and apoptosis in cocultured FaDu and NPC‐039 cells (Figures 4A–D and 5A–D). In FaDu cells, the overall percentage of apoptotic cells in the treatment group (FaDu cells cocultured with KHYG‐1 cells) increased to 25.74%, 28.3%, and 31.04% at different concentrations of quinovic acid (Figure 4C,D). In NPC‐039 cells, the overall percentage of apoptotic cells in the treatment group increased to 25.95%, 29.75%, and 33.77% at different concentrations of quinovic acid (Figure 5C,D). In addition, we examined the expression of cytotoxicity‐related proteins in FaDu and NPC‐039 cells. Quinovic acid significantly increased the expression of Fas and the cleaved forms of t‐Bid, caspase‐3, ‐8, ‐9, and PARP, while reducing the levels of SET, Ape1, and HMGB2 in cocultured target cells (Figures 4E–H and 5E–H). These findings indicate that quinovic acid enhances KHYG‐1‐mediated cytotoxicity, leading to apoptosis in diverse cancer cell types, including K562, FaDu, and NPC‐039.

### 3.4 Effect of Quinovic Acid on MAPK Signalling Pathways

**FIGURE 6:**
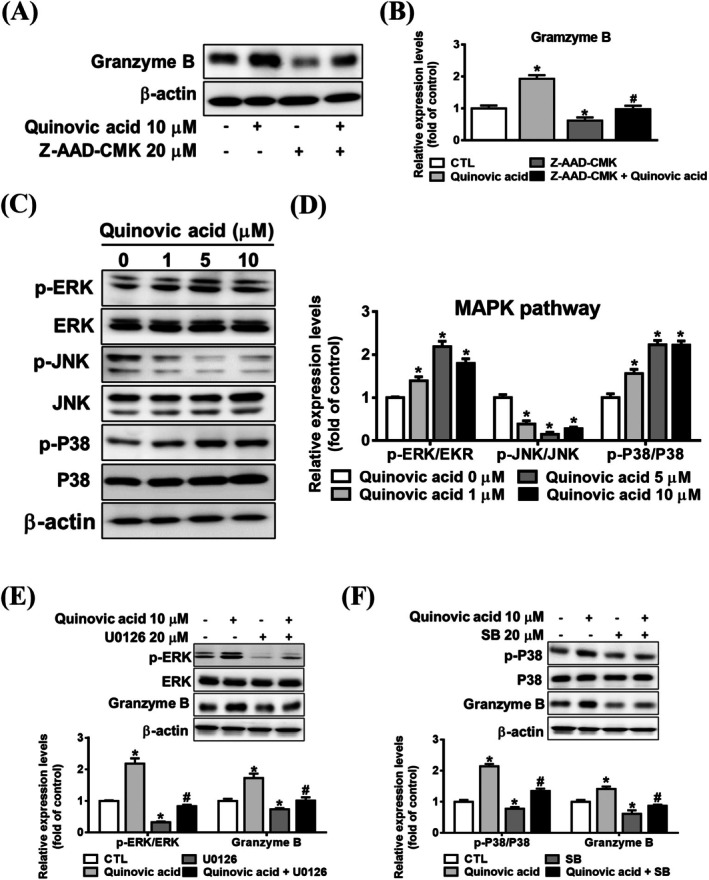
Quinovic acid induced granzyme B expression through the MAPK pathway. (A, B) KHYG‐1 cells were pretreated with granzyme B inhibitor Z‐AAD‐CMK for 1 h, followed by cotreatment with 10 μM quinovic acid for 24 h. Western blot analysis was performed to measure granzyme B protein expression. (C, D) Cells were treated with quinovic acid (0–10 μM) for 24 h, and MAPK pathway protein expression was analysed. (E, F) Cells were pretreated with MAPK inhibitors (U0126 for ERK and SB203580 for P38) for 1 h or left untreated and then treated with quinovic acid for 24 h. Protein levels were quantified using Western blotting, normalised to β‐actin, and analysed using a densitometer. *p < 0.05 compared with control; ^#^ *p* < 0.05 compared with inhibitor‐treated cells.

To elucidate the mechanism through which quinovic acid increases granzyme B expression, we used Z‐AAD‐CMK, a granzyme B inhibitor, as a pretreatment. Cotreatment with Z‐AAD‐CMK substantially reduced granzyme B protein expression (Figure 5A,B). The MAPK signalling pathway regulates the transcription and expression of granzyme B [26]. To confirm the involvement of the MAPK pathway in quinovic acid–induced granzyme B expression, we examined the phosphorylation levels of ERK, P38, and JNK. Quinovic acid substantially increased the phosphorylation of ERK and P38 but not JNK (Figure 6C,D). In addition, we cotreated KHYG‐1 cells with quinovic acid and specific inhibitors (U0126 for ERK and SB for P38). Our data indicated that cotreatment with these inhibitors reduced the levels of p‐ERK, p‐P38, and granzyme B compared with treatment with quinovic acid alone in KHYG‐1 cells (Figure 6E,F). Taken together, these findings suggest that quinovic acid upregulates granzyme B expression through the phosphorylation of ERK and P38.

### 3.5 Quinovic Acid–Induced Activation of NKR Signalling Leads to Increased IFN‐γ Expression in NK Cells

**FIGURE 7:**
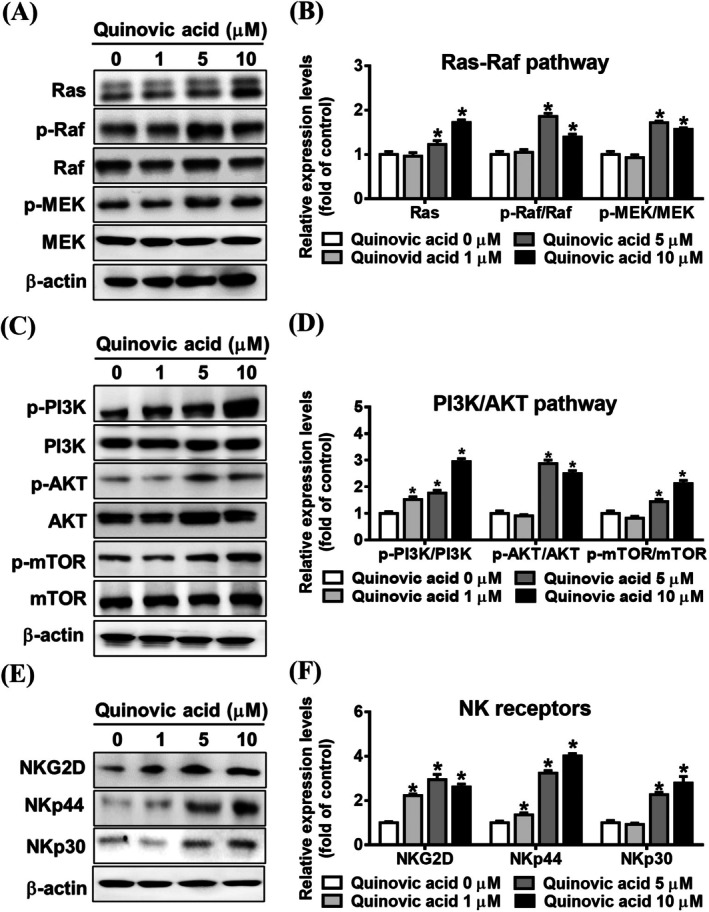
Quinovic acid increased NK receptor expression and activated the Ras–Raf pathway. KHYG‐1 cells were treated with quinovic acid (0–10 μM) for 24 h. Protein expression levels were analysed using Western blotting. (A, B) Expression of proteins in the Ras–Raf pathway. (C, D) Expression of proteins in the PI3K/AKT pathway. (E, F) Expression of NK receptors. Protein quantification was performed using ImageJ, with all protein levels normalised to that of β‐actin. Data are presented as the mean ± standard deviation. *p < 0.05 compared with control.

Activation of IFN‐γ production in human NK cells through NKG2D and other NK receptors involves the MAPK and PI3K/AKT pathways [27]. We investigated the association between quinovic acid and IFN‐γ expression. After the treatment of KHYG‐1 cells with quinovic acid, we analysed the expression of Ras/Raf/MEK (Figure 7A,B) and PI3K/AKT (Figure 7C,D). Quinovic acid substantially increased the phosphorylation levels of these proteins in KHYG‐1 cells. In addition, quinovic acid increased the expression of the activating receptors NKp44, NKp30, and NKG2D (Figure 7E,F).

**FIGURE 8:**
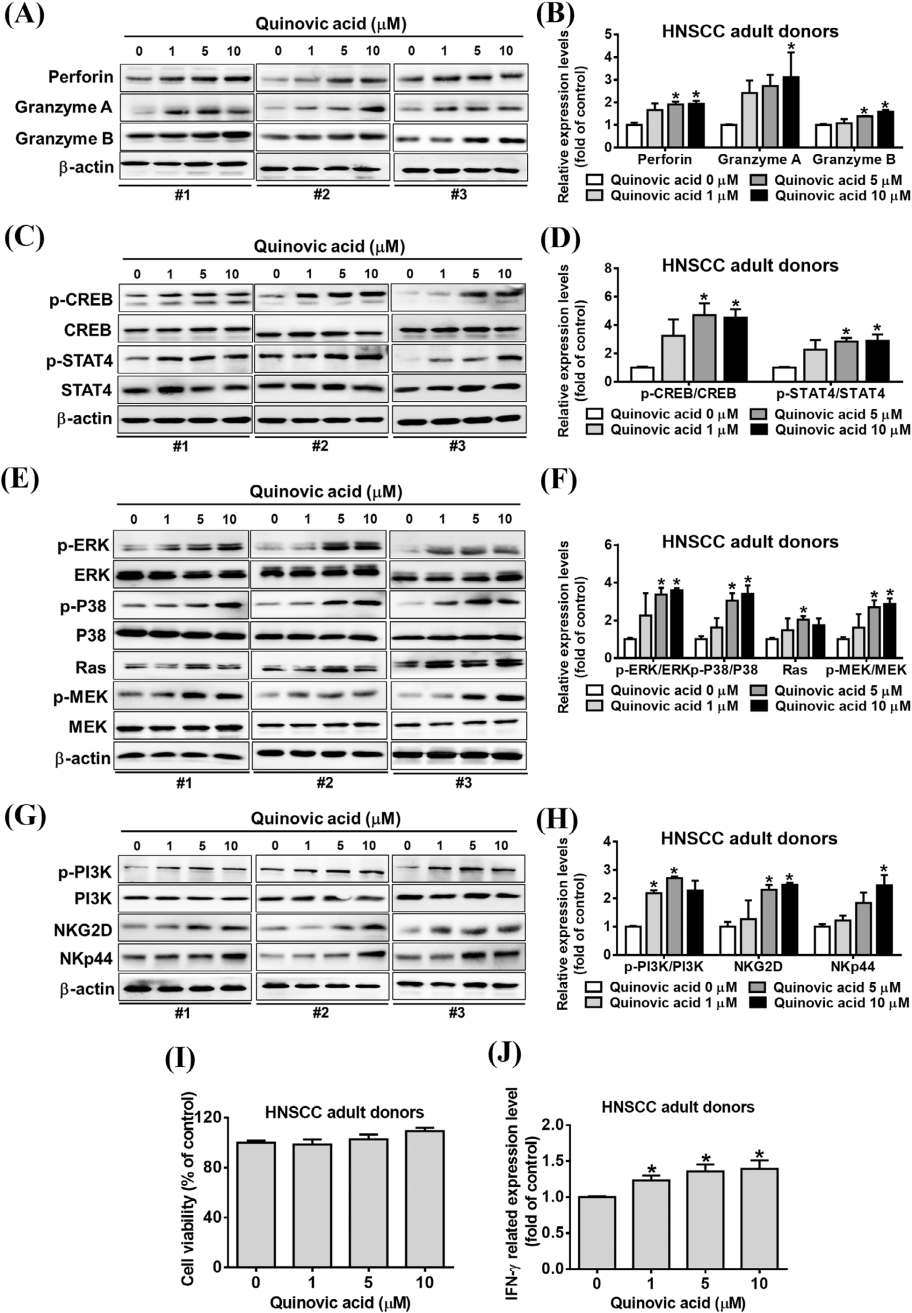
Effects of quinovic acid treatment on NK cells derived from adult patients with head and neck squamous cell carcinoma. NK cells were treated with quinovic acid for 24 h. Protein expression levels were analysed using Western blotting. (A, B) Key proteins associated with cytolytic activity in NK cells. (C, D) Transcription factors. (E, F) Ras and MAPK pathways. (G, H) NK receptors. Protein quantification was performed using ImageJ, with all protein levels normalised to that of β‐actin. Data are presented as the mean ± standard deviation. *p < 0.05 compared with control. (I) Results of the WST‐8 assay performed to evaluate the viability of NK cells treated with quinovic acid for 24 h. (J) IFN‐γ level was measured using an enzyme‐linked immunosorbent assay following 24‐h treatment with quinovic acid.

We investigated the effect of quinovic acid on NK cells derived from three adult patients with head and neck squamous cell carcinoma to determine whether quinovic acid enhances cytotoxicity in other cancer cell lines. Initially, we determined that NK cells obtained from human donors did not exert a cytotoxic effect under the same treatment conditions (Figure 8I). Quinovic acid significantly increased IFN‐γ expression in these NK cells (Figure 8J). The results of Western blot analysis indicated that quinovic acid increased the release of cytotoxic granules, including granzyme A and B (Figure 8A,B), and enhanced the phosphorylation of transcription factors (Figure 8C,D). The activation of the NK receptors NKp44, NKp30, and NKG2D led to increased IFN‐γ secretion, which was associated with the MAPK and PI3K/AKT pathways (Figure 8E–H). These results indicated that quinovic acid enhanced the cytotoxicity of not only KHYG‐1 cells but also NK cells derived from adult patients with head and neck squamous cell carcinoma. We repeated the same experiments by using NK cells obtained from healthy adult donors, and similar results were observed (Figure S1).

## 4 Discussion

NK cell–based therapy has been explored as a tool against cancer for decades. Clinical trials have demonstrated the safety of NK cell infusion, even in allogeneic settings. However, challenges remain, such as substantially expanding primary NK cells, maintaining their persistence in vivo, and enhancing their cytotoxicity against tumours [11]. In the present study, we observed that quinovic acid, a natural triterpene, enhanced the cytotoxicity of KHYG‐1 cells. Quinovic acid increased the expression of various cytolytic proteins, enhanced the cytotoxicity of KHYG‐1 cells against K562 cells, and induced the secretion of cytotoxic cytokines (e.g., INF‐γ; Figure 1) without affecting KHYG‐1 cell proliferation. Then, we elucidated the mechanism through which quinovic acid induced granzyme B expression. We found that quinovic acid increased histone acetylation; this effect was more pronounced when quinovic acid was combined with TSA (Figure 2C–F). However, combining it with TSA did not result in increased granzyme B expression (data not shown). Similar results were observed in KHYG‐1 cells treated with nobiletin and raddeanin A [5, 22]. CREB regulates the transcription of both perforin and granzyme B genes [28]. Phosphorylated STAT4 is a key signalling pathway that leads to IFN‐γ transcription [27, 29]. The results of the present study indicate that quinovic acid activates granzyme B and perforin transcription by increasing the level of phosphorylated CREB (Figure 2A,B).

In the current study, we cocultured KHYG‐1 and K562 cells and found that quinovic acid stimulated the release of cytotoxic granules, inducing apoptosis signalling pathways in K562 cells. The cytotoxic granule granzyme B activates caspase‐3 and induces the release of cytochrome c from the mitochondria after cleaving the Bid protein, leading to apoptosis through a caspase‐dependent pathway [20, 30]. By contrast, granzyme A induces apoptosis through a caspase‐independent pathway by cleaving the SET complex upon entry into the nucleus. The SET complex consists of three nucleases (SET, Ape1, and HMGB2) and plays a role in DNA repair [31, 32]. In our results (Figure 1), quinovic acid induced an increase in granzyme A protein expression in KHYG‐1 cells. Under coculture conditions (Figures 3, 4, 5), we observed a decrease in the expression of SET complex proteins. Previous studies have reported that granzyme A cleaves NDUFS3, a component of the electron transport chain, thereby generating reactive oxygen species (ROS). ROS are responsible for initiating downstream events, such as nuclear translocation of the SET complex, providing immune cells with a mechanism to eliminate targets resistant to other forms of programmed cell death [32, 33]. We further tested whether quinovic acid induces target cell death through an ROS‐mediated mechanism. As shown in Figure S2, quinovic acid alone did not alter ROS levels in either KHYG‐1 cells or target cells (K562, FaDu, and NPC‐039). However, in target cells cocultured with KHYG‐1 cells, quinovic acid treatment significantly increased ROS levels (percentage) (Figure S2). The cytotoxic cytokine IFN‐γ regulates the Fas/FasL signalling pathway [34], which subsequently activates caspase‐8 and triggers apoptosis [35]. Our findings are consistent with those of other studies (Figure 3). Quinovic acid enhanced KHYG‐1 cell–mediated cytotoxicity, resulting in the loss of mitochondrial membrane potential and the induction of apoptosis in cocultured K562 cells. The expression of apoptosis‐related proteins in target cells indicated the activation of both caspase‐dependent (t‐Bid, cleaved caspase‐3, and cleaved PARP) and caspase‐independent (SET, Ape1, and HMGB2) pathways, along with caspase‐8 activation and upregulation of the Fas/FasL signalling pathway. Similar KHYG‐1–mediated apoptotic effects were also observed when cocultured with other cancer cell types, including FaDu and NPC‐039 cells (Figures 4 and 5).

Granzyme B plays a crucial role as a protein in cytotoxic granules released by NK cells. We examined the role of the MAPK pathway in regulating granzyme B expression (Figure 6). Quinovic acid substantially increased the phosphorylation of ERK and P38 but not JNK. Cotreatment of KHYG‐1 cells with MAPK pathway inhibitors (U0126 for ERK and SB for P38) reduced the expression of granzyme B in KHYG‐1 cells compared with treatment with quinovic acid alone. Quinovic acid increased granzyme B expression in KHYG‐1 cells by upregulating ERK/P38 phosphorylation. This result is consistent with those of other studies [5, 19]. Ginsenoside 20(R)‐Rg3 enhances NK cell activity through the ERK signalling pathway without affecting P38 or JNK phosphorylation [36]. Although quinovic acid reduced JNK phosphorylation in the present study, this reduction did not influence the expression of granzyme B or the cytotoxic activity of KHYG‐1 cells.

Quinovic acid enhanced IFN‐γ secretion from KHYG‐1 cells (Figure 1D). We explored mechanisms underlying this upregulation of IFN‐γ. NK cells mainly produce IFN‐γ through three coordinated pathways: (1) activation of NK receptors, which leads to protein kinase activation (such as MAPK, PI3K/AKT, and PLC‐γ); (2) antibody‐dependent recognition, involving antigen‐presenting cells or coculture with tumour cells; and (3) stimulation by immune cytokines (e.g., IL‐2 and IL‐12), with IL‐12 co‐stimulation and STAT4 activation specifically inducing IFN‐γ transcription [27, 37, 38, 39, 40]. The findings of the present study (Figure 7) revealed that quinovic acid treatment increased the expression of NKG2D and NKRs (NKp44 and NKp30) and subsequently activated the Ras/Raf, MEK/MAPK, and PI3K/AKT phosphorylation signalling pathways. We also observed an increase in STAT4 phosphorylation. Our results indicate the presence of multiple pathways through which quinovic acid enhances IFN‐γ secretion in KHYG‐1 cells. We also explored the LLT1 receptor that promotes IFN‐γ secretion in NK cells through Ras/Raf and MAPK signalling pathways [41, 42]. LLT1 expression remained unchanged after quinovic acid treatment (data not shown). The PI3K/AKT pathway is essential for the proliferation and activation of NK cells [43, 44]. Quinovic acid treatment for 72 h did not affect KHYG‐1 cell proliferation (Figure 1B).

In the present study, quinovic acid enhanced the cytotoxicity of NK cells derived from adult patients with head and neck squamous cell carcinoma, demonstrating effects comparable to those observed in KHYG‐1 cells. In conclusion, quinovic acid significantly increases the expression of cytotoxic granules, IFN‐γ, and NK cell receptors and activates the Ras/Raf and PI3K/AKT signalling pathways. Similarly, in NK cells from healthy donors, quinovic acid increases cytotoxicity through the same signalling pathways (Figure S1).

## 5 Conclusion

Quinovic acid, a natural triterpene, enhanced the cytotoxic activity of KHYG‐1 cells against K562 cells by upregulating CREB‐mediated expression of cytolytic proteins. In coculture experiments with K562, FaDu, and NPC‐039 cells, quinovic acid indirectly induced ROS levels, mitochondrial membrane depolarisation and apoptosis in target cells through KHYG‐1‐mediated cytotoxicity involving both caspase‐dependent and caspase‐independent pathways. Mechanistic analysis revealed that quinovic acid increased ERK and P38 phosphorylation, which in turn upregulated granzyme B expression in KHYG‐1 cells. Furthermore, quinovic acid promoted IFN‐γ expression by upregulating NK receptors and activating the STAT4, PI3K/AKT, and Ras/Raf signalling pathways. Consistent results were observed when primary human NK cells were used.

## Author Contributions

**Ming‐Ju Hsieh:** conceptualization (equal), writing – original draft (equal), writing – review and editing (equal). **Jen‐Tsun Lin:** conceptualization (equal), writing – review and editing (equal). **Yi‐Ching Chuang:** methodology (equal), software (equal), writing – original draft (equal). **Hsin‐Yu Ho:** methodology (equal), software (equal), writing – review and editing (equal). **Yu‐Sheng Lo:** methodology (equal), software (equal). **Chia‐Chieh Lin:** methodology (equal), software (equal). **Mu‐Kuan Chen:** conceptualization (equal), writing – review and editing (equal).

## Funding

This study was supported by grants from the Changhua Christian Hospital, Changhua City, Taiwan (113‐CCH‐IRP‐005).

## Ethics Statement

This study's protocol was approved by the Institutional Review Board (IRB) of the Changhua Christian Hospital (Changhua, Taiwan; IRB No. 230602, date of approval: 11 July, 2023).

## Consent

Informed consent was obtained from all subjects involved in the study.

## Conflicts of Interest

The authors declare no conflicts of interest.

## Supporting information


**Figure S1:** Effects of quinovic acid treatment on NK cells derived from healthy adult donors. NK cells were treated with quinovic acid for 24 h. Protein expression levels were analysed using Western blotting. (A, B) Key proteins associated with cytolytic activity and transcription factors in NK cells. (C, D) Ras and MAPK pathways. (E, F) NK receptors. Protein quantification was performed using ImageJ, with all protein levels normalised to that of β‐actin. Data are presented as the mean ± standard deviation. *p* < 0.05 compared with control. (G) Results of the WST‐8 assay performed to assess the viability of NK cells treated with quinovic acid for 24 h. (H) IFN‐γ level was measured using an enzyme‐linked immunosorbent assay following 24‐h treatment with quinovic acid.

**Figure S2:** Used KHYG‐1 cell cocultured with target cells to analysed the quinovic acid‐induced Reactive Oxygen Species (ROS). (A) The cells (KHYG‐1, K562, FaDu, and NPC‐039) were treated with quinovic acid 10 μM or H_2_O_2_ 100 μM (positive control) for 24 h. ROS levels were assessed using the Muse Oxidative Stress Kit. (B) Quantitative data were analysed using Muse Cell Software V1.4.0.0. (C) K562, FaDu, and NPC‐039 were cocultured with KHYG‐1 cells at an effector: target ratio of 6:1 and then treated with quinovic acid (0–10 μM) or H_2_O_2_ 100 μM for 24 h. Compared with control cells, statistically significant results were obtained in treated cells. *p < 0.05 vs. control, ^#^ *p* < 0.05 vs. cotreatment control.

## Data Availability

The data used to support the findings of this study are available from the corresponding author upon request.
