# Supplementary material for: Quinovic Acid Enhances the Cytotoxicity of KHYG‐1 Cells by Modulating the Ras/MAPK Signalling Pathway and Interferon‐Gamma Expression
Source: J Cell Mol Med. 2025 Nov 26;29(22):e70957. doi: 10.1111/jcmm.70957 (PMC12648300; doi:10.1111/jcmm.70957)
Supplement: Supplementary file 1 — Figure S1: Effects of quinovic acid treatment on NK cells derived from healthy adult donors. NK cells were treated with quinovic acid for 24 h. Protein expression levels were analysed using Western blotting. (A, B) Key proteins associated with cytolytic activity and transcription factors in NK cells. (C, D) Ras and MAPK pathways. (E, F) NK receptors. Protein quantification was performed using ImageJ, with all protein levels normalised to that of β‐actin. Data are presented as the mean ± standard deviation. p < 0.05 compared with control. (G) Results of the WST‐8 assay performed to assess the viability of NK cells treated with quinovic acid for 24 h. (H) IFN‐γ level was measured using an enzyme‐linked immunosorbent assay following 24‐h treatment with quinovic acid. [file JCMM-29-e70957-s001.docx]

**Figure S1**


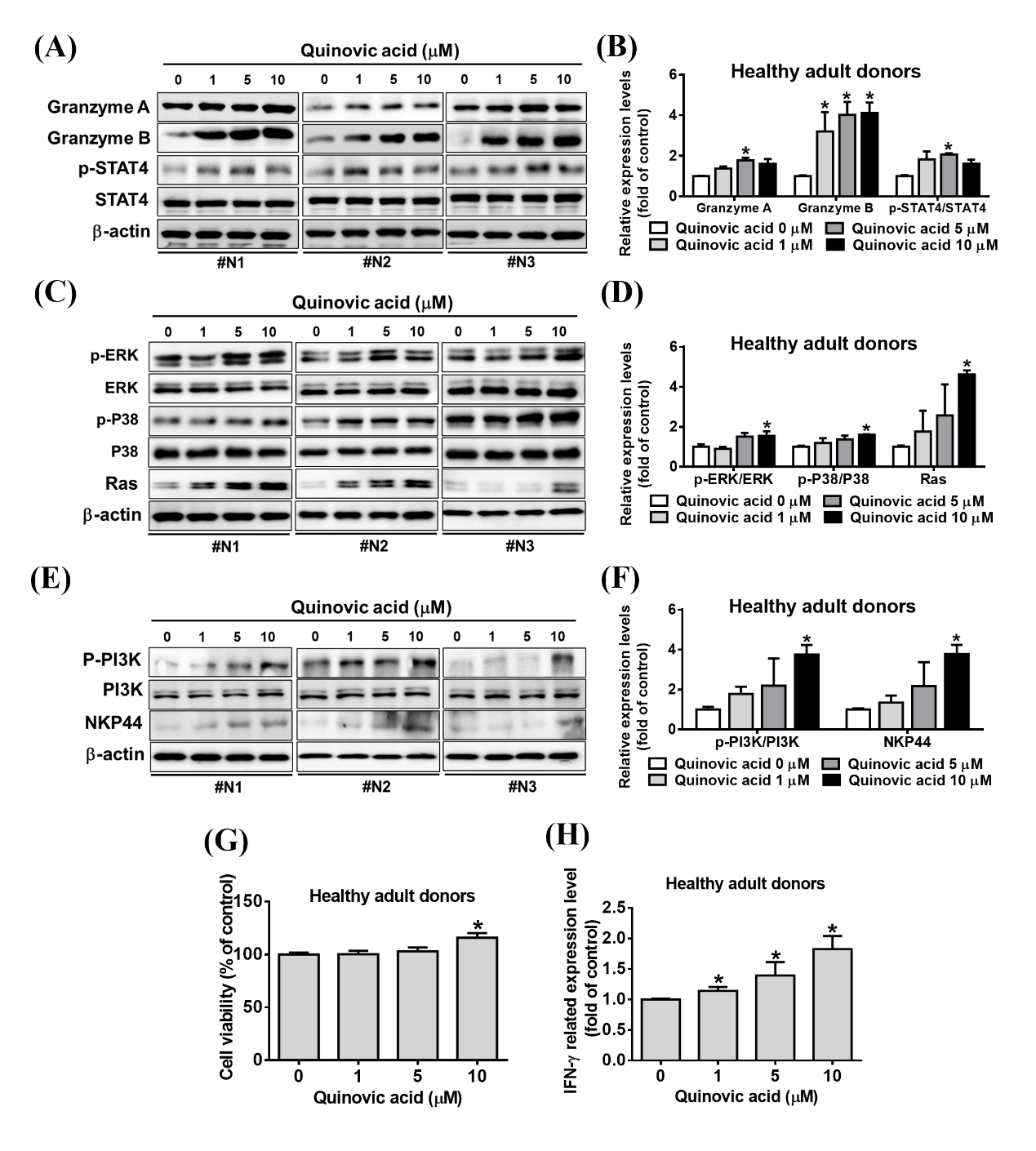


**Figure S1. Effects of quinovic acid treatment on NK cells derived from healthy adult donors**

NK cells were treated with quinovic acid for 24 h. Protein expression levels were analyzed using Western blotting. (A, B) Key proteins associated with cytolytic activity and transcription factors in NK cells. (C, D) Ras and MAPK pathways. (E, F) NK receptors. Protein quantification was performed using ImageJ, with all protein levels normalized to that of β-actin. Data are presented as the mean ± standard deviation. *p* < 0.05 compared with control. (G) Results of the WST-8 assay performed to assess the viability of NK cells treated with quinovic acid for 24 h. (H) IFN-γ level was measured using an enzyme-linked immunosorbent assay following 24-h treatment with quinovic acid.
