# Supplementary material for: Quinovic Acid Enhances the Cytotoxicity of KHYG‐1 Cells by Modulating the Ras/MAPK Signalling Pathway and Interferon‐Gamma Expression
Source: J Cell Mol Med. 2025 Nov 26;29(22):e70957. doi: 10.1111/jcmm.70957 (PMC12648300; doi:10.1111/jcmm.70957)
Supplement: Supplementary file 2 — Figure S2: Used KHYG‐1 cell cocultured with target cells to analysed the quinovic acid‐induced Reactive Oxygen Species (ROS). (A) The cells (KHYG‐1, K562, FaDu, and NPC‐039) were treated with quinovic acid 10 μM or H2O2 100 μM (positive control) for 24 h. ROS levels were assessed using the Muse Oxidative Stress Kit. (B) Quantitative data were analysed using Muse Cell Software V1.4.0.0. (C) K562, FaDu, and NPC‐039 were cocultured with KHYG‐1 cells at an effector: target ratio of 6:1 and then treated with quinovic acid (0–10 μM) or H2O2 100 μM for 24 h. Compared with control cells, statistically significant results were obtained in treated cells. *p < 0.05 vs. control, # p < 0.05 vs. cotreatment control. [file JCMM-29-e70957-s002.docx]

**Figure S2**

**
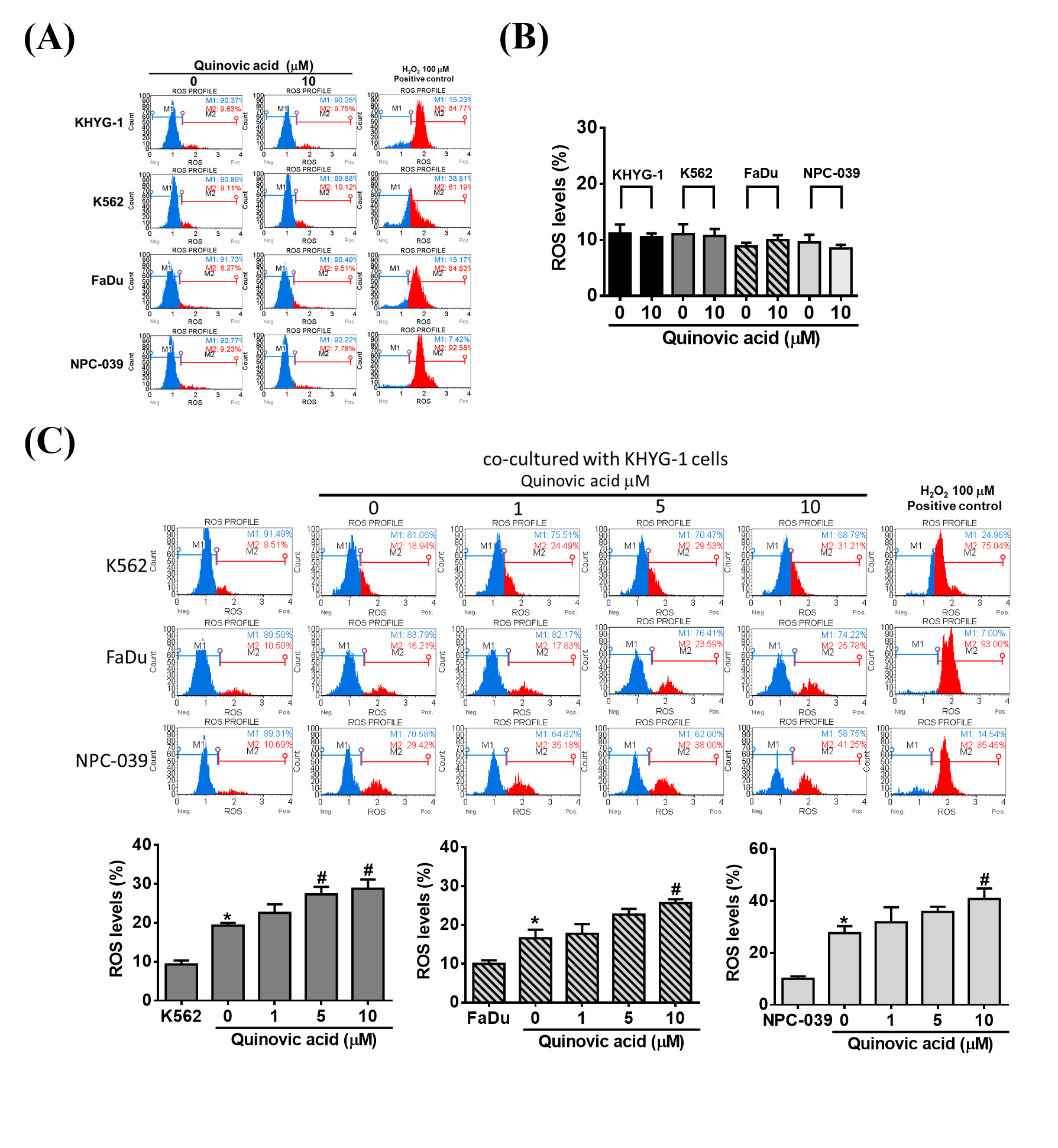
**

**Figure S2. Used KHYG-1 cell cocultured with target cells to analyzed the quinovic acid-induced Reactive Oxygen Species (ROS)**

(A) The cells (KHYG-1, K562, FaDu, and NPC-039) were treated with quinovic acid 10 μM or H_2_O_2_ 100 μM (positive control) for 24 h. ROS levels were assessed using the Muse Oxidative Stress Kit. (B) Quantitative data were analyzed using Muse Cell Software V1.4.0.0. (C) K562, FaDu, and NPC-039 were cocultured with KHYG-1 cells at an effector : target ratio of 6:1 and then treated with quinovic acid (0-10 μM) or H_2_O_2_ 100 μM for 24 h. Compared with control cells, statistically significant results were obtained in treated cells. **p* < 0.05 vs. control, # *p* < 0.05 vs. cotreatment control

**Materials and Methods**

**Reactive Oxygen Species measurement**

KHYG-1 cells were treated with indicated doses of quinovic acid and added to the upper wells of a transwell insert (Greiner Bio-One, Monroe, NC, USA). K562 or FaDu or NPC-039 cells (in RPMI-1640 media) were added to the lower wells of the same insert and incubated. The effector cells were cocultured with the target cells at an effector: target (E: T) ratio of 6:1 for 24 h at 37℃ under 5% CO_2_. Then the target cells collected and stained with Muse Oxidative Stress Kit for 30 min at 37℃, and measurement of experimental signals was done by Muse Cell Analyzer flow cytometry (Merck Millipore, Burlington, MA, USA), and the data were analyzed by Muse Cell Soft V1.4.0.0.
